# Subject specificity of the correlation between large-scale structural and functional connectivity

**DOI:** 10.1162/netn_a_00055

**Published:** 2018-10-01

**Authors:** J. Zimmermann, J. Griffiths, M. Schirner, P. Ritter, A. R. McIntosh

**Affiliations:** Baycrest Health Sciences, Rotman Research Institute, Toronto, Ontario, Canada; Baycrest Health Sciences, Rotman Research Institute, Toronto, Ontario, Canada; Department of Neurology, Charité–Universitätsmedizin Berlin, Corporate Member of Freie Universitët Berlin, Humboldt-Universität zu Berlin, and Berlin Institute of Health; Bernstein Focus State Dependencies of Learning & Bernstein Center for Computational Neuroscience, Berlin, Germany; Department of Neurology, Charité–Universitätsmedizin Berlin, Corporate Member of Freie Universitët Berlin, Humboldt-Universität zu Berlin, and Berlin Institute of Health; Bernstein Focus State Dependencies of Learning & Bernstein Center for Computational Neuroscience, Berlin, Germany; Minerva Research Group BrainModes, Max Planck Institute for Human Cognitive and Brain Sciences, Leipzig, Germany; Berlin School of Mind and Brain & Mind and Brain Institute, Humboldt University, Berlin, Germany; Berlin Institute of Health (BIH), Berlin, Germany; Baycrest Health Sciences, Rotman Research Institute, Toronto, Ontario, Canada

**Keywords:** Structural connectivity, Functional connectivity, Individual variability

## Abstract

Structural connectivity (SC), the physical pathways connecting regions in the brain, and functional connectivity (FC), the temporal coactivations, are known to be tightly linked. However, the nature of this relationship is still not understood. In the present study, we examined this relation more closely in six separate human neuroimaging datasets with different acquisition and preprocessing methods. We show that using simple linear associations, the relation between an individual’s SC and FC is not subject specific for five of the datasets. Subject specificity of SC-FC fit is achieved only for one of the six datasets, the multimodal Glasser Human Connectome Project (HCP) parcellated dataset. We show that subject specificity of SC-FC correspondence is limited across datasets due to relatively small variability between subjects in SC compared with the larger variability in FC.

## Author Summary

We present evidence that, in most standard datasets, the subject variation in structural connectivity (SC) may be too weak to be reflected in the functional connectivity (FC) variability. However, subject specificity of SC-FC can be captured via fine, multimodally parcellated data because of greater SC variability across subjects. Nonetheless, SC and FC each show a large component that is common across subjects, which sets limitations on the extent of SC-FC subject specificity. Implications of these findings for personalized medicine should be considered. Namely, attention to the quality of processing and parcellation methods is critical for furthering our understanding of the relationship between individual SC and FC.

## INTRODUCTION

It has been shown that there is a relationship between structural connectivity (SC), the physical white matter tracts between regions, and resting-state functional connectivity (FC), the temporal coactivations between regions (Greicius, Supekar, Menon, & Dougherty, 2009; Hermundstad et al., 2013; Honey, Kotter, Breakspear, & Sporns, 2007; Honey et al., 2009; Koch, Norris, & Hund-Georgiadis, 2002; Misic et al., 2016; Ponce-Alvarez et al., 2015; Skudlarski et al., 2008; van den Heuvel, Mandl, Kahn, & Hulshoff Pol, 2009; van den Heuvel & Sporns, 2013) usingboth simple linear (Honey et al., 2009) as well as more complex metrics (Misic et al., 2016). Most of this research, however, considers group-averaged matrices of SC and FC rather than individual connectomes. Motivated by the recent interest in personalized medicine and precision science, there is a greater need to understand individual differences and unique relationships between SC and FC. One important question is whether individual SC correlates with the corresponding subject’s FC to a greater extent than between subjects. Correlations between whole-brain individual SC and FC have been associated with measures of behavior or clinical conditions (Caeyenberghs, Leemans, Leunissen, Michiels, & Swinnen, 2013; Cocchi et al., 2014; Skudlarski et al., 2010; Zhang et al., 2011). Yet, there are very few studies that investigate the subject specificity of this SC-FC correspondence (Honey et al., 2009; Meier et al., 2016), and as far as we know there are no studies that assert that individual SC maps best onto its corresponding FC by using linear measures of association. One preliminary investigation conducted by Honey et al. (2009) examined this question; however, results were inconclusive due to the limited sample size. Clearly, it is not well understood whether there is a unique portion of variance in SC accounting for unique individual differences in FC.

It has already been shown that individual structural and functional connectomes can be sensitive to age (Zimmermann et al., 2016), personality traits (Markett et al., 2013), as well as cognition, demographics, and behavior (Hearne, Mattingley, & Cocchi, 2016; Ponsoda et al., 2017; S. Smith, 2016; S. M. Smith et al., 2015). Moreover, SC (Kumar, Desrosiers, Siddiqi, Colliot, & Toews, 2017; Munsell et al., 2017; Yeh et al., 2016) as well as FC (Amico & Goñi, 2017; Finn et al., 2015) can be used to identify individual connectome fingerprints. Nonetheless, the extent of this individual variability has been called into question (Marrelec, Messe, Giron, & Rudrauf, 2016; Waller et al., 2017), particularly for smaller sample sizes (Waller et al., 2017). For instance, it has been shown that variability in FC can be explained by only one or two dimensions, and that FC is highly degenerate in its ability to capture potential complexities and variability in underlying dynamics (Marrelec et al., 2016).

Variance decomposition methods, such as principal components analysis (PCA), are helpful for characterizing the strength of individual differences across connectomes (Amico & Goñi, 2017; Marrelec et al., 2016). PCA provides a simplified representation of the data by reducing the existing variance into a smaller number of components. In this way, the portion of variance that is common across subjects can be identified and separated from the unique aspects of variance.

The aim of the present study was to investigate the subject specificity of the SC-FC relationship. The analyses were conducted on six datasets with variable acquisition schemes, preprocessing methods, and sample sizes (*N* = 48, 626, 171, 766, 754, 754). Four of these were variations of HCP data (Van Essen et al., 2013). We used simple linear measures of association with bootstrapping to quantify the correspondence of within-subject and between-subject SC-FC, and decomposition to quantify the extent of common and unique variability in SC and FC across subjects.

## METHODS

### Data Acquisition and Preprocessing

The analyses were conducted on six MRI datasets of healthy subjects: the Berlin dataset (*N* = 48; Ritter, Schirner, McIntosh, & Jirsa, 2013; Schirner, Rothmeier, Jirsa, McIntosh, & Ritter, 2015; Zimmermann et al., 2016), the Nathan Kline Institute (NKI) Rockland dataset from the UMCD Multimodal connectivity database (*N* = 171; Brown, Rudie, Bandrowski, Van Horn, & Bookheimer, 2012), and four variations from the HCP dataset (S900 release; Van Essen et al., 2013) that differed in terms of processing methods as well as parcellation schemes. These were the HCP Lausanne dataset (*N* = 626), HCP Glasser dataset (*N* = 766), HCP Destrieux dataset (*N* = 754), and HCP Desikan-Killiany (DK) dataset (*N* = 754). Note that sample size differences between HCP datasets were due to removal of subjects with problematic parcellations. The HCP Glasser dataset was parcellated via a high-resolution multimodal scheme based on an areal feature-based cross-subjects alignment method (Glasser et al., 2016). The research was performed in compliance with the Code of Ethics of the World Medical Association (Declaration of Helsinki). Written, informed consent was provided by all subjects with an understanding of the study prior to data collection, and was approved by the local ethics committee in accordance with the institutional guidelines at Charité Hospital, Berlin, UCLA, and HCP WU-Minn.

A detailed description of data acquisition procedures is presented in Supporting Information Table S1 (Zimmermann, Griffiths, Schirner, Ritter, & McIntosh, 2018). Subject sample size, age range, processing, and parcellation information are presented in Table 1, along with references to previously published papers with these datasets. Quality control was described in detail in those papers. For the Berlin and NKI Rockland dataset, noise correction was performed via nuisance variable regression from the BOLD signal, including six motion parameters, mean white matter, and CSF signals. For the HCP datasets, we used FIX-denoised data, a tool that was trained to effectively remove components of the white matter, CSF, physiological noise, and 24 high-pass-filtered motion parameters from the signal (Glasser et al., 2013).

**Table 1.** Dataset details for six MRI diffusion and resting-state datasets, including sample sizes, preprocessing methods, and parcellation schemes

	**Berlin**	**NKI Rockland**	**HCP, Lausanne**	**HCP, Glasser**	**HCP, Destrieux**	**HCP, DK**
Processing reference	Schirner et al., 2015	Brown et al., 2012	Glasser et al., 2013	Glasser et al., 2013	Glasser et al., 2013	Glasser et al., 2013
Sample size	48	171	626	766	754	754
Subject ages	18–80	5–85	22–36	22–37	22–37	22–37
	*M* = 41.90	*M* = 35.80	*M* = 28.65	*M* = 28.78	*M* = 28.78	*M* = 28.78
	*SD* = 18.47	*SD* = 19.99	*SD* = 3.66	*SD* = 3.70	*SD* = 3.70	*SD* = 3.70
Parcellation (ROIs)	Desikan-Killiany (68) (Desikan et al., 2006)	Craddock (188)	Lausanne (83) (Daducci et al., 2012)	Glasser (378) (Glasser et al., 2016)	Destrieux (164) (Destrieux, Fischl, Dale, & Halgren, 2010)	Desikan-Killiany (84) (Desikan et al., 2006)

**Structural and diffusion processing**						
Software method	FreeSurfer	FreeSurfer & Dipy	TrackVis Diffusion Toolkit	HCP pipeline (Glasser et al., 2013)	HCP pipeline (Glasser et al., 2013)	HCP pipeline (Glasser et al., 2013)
Motion/eddy correction	yes	yes	yes	yes	yes	yes
Intensity normalization	yes	no	yes	yes	yes	yes
Tractography	Probabilistic (MRTrix)	Deterministic (FACT)	Deterministic (EuDX)	Probabilistic (MRTrix)	Probabilistic (MRTrix)	Probabilistic (MRTrix)
SC metric	Voxel pairs connected w streamline, ROI volume corrected	Streamline count	Streamline count	Weighted streamline count (SIFT2) by cross-sectional area	Weighted streamline count (SIFT2)	Weighted streamline count (SIFT2)

**Functional processing**						
Software method	Schirner et al., 2015	fMRI FEAT	Glasser et al., 2013	Glasser et al., 2013	Glasser et al., 2013	Glasser et al., 2013
Slice-timing	no	yes	no	no	no	no
Motion correction	MCFLIRT	MCFLIRT	FIX denoise	6 DOF FLIRT FIX denoise	6 DOF FLIRT FIX denoise	6 DOF FLIRT FIX denoise
Nuisance regression	6 motion, mean WM, CSF	24 motion, mean WM, CSF, volume	no	no	no	no
Smoothing	no	FWHM 5 mm Gaussian	no	Cort surf, subcort vol, FWHM 2 mm Gaussian	Cort surf, subcort vol, FWHM 2 mm Gaussian	Cort surf, subcort vol, FWHM 2 mm Gaussian
Intensity normalization	no	yes	no	yes	yes	yes
Temporal filtering	High-pass 100 s	Band-pass 0.08–0.009 Hz	no	no	no	no
Registration to standard space	no	MNI152	no	MNI152 & surf-based multimodal (MSMAll, Robinson et al., 2014)		
Motion scrub	no	yes	no	no	no	no

SC and FC were derived via diffusion-weighted magnetic resonance imaging (dwMRI) and resting-state blood oxygen dependent functional magnetic resonance imaging (rsfMRI BOLD), respectively. Structural and functional data were parcellated into predefined regions of interest (ROIs) that varied in size across datasets (68–378 cortical regions). Fiber track estimation was performed on the diffusion data, and weight and distance SCs were computed by aggregating tractography-based estimations of white matter streamlines between ROIs. Each entry in the SC weights matrix was an estimate of the connection strength between a pair of ROIs. SC distances were the Euclidian distances (Brown et al., 2012; Glasser et al., 2013; Hagmann et al., 2008) or average length of tracks (Schirner et al., 2015) in millimeters between pairs of ROIs. We corrected for SC distance by regressing distances from weight SCs and using residuals for analysis (as tract length may have an effect on structure-function relations; Romero-Garcia, Atienza, & Cantero, 2014). To account for age-related differences in parcellation and ROI size in the Berlin dataset, SCs were weighted by the mean gray-matter white-matter interface area of connected ROIs. FCs were computed as the Pearson’s correlation between each ROI pair of BOLD time series, and were transformed to a normal distribution via a Fisher’s *r* to *z*.

### Subject Specificity of SC-FC Predictions

**Figure 1.**
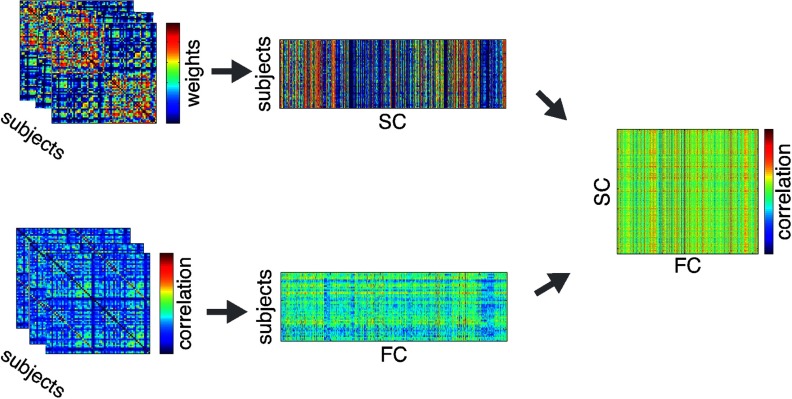
Individual subject SCs and FCs were stacked into a subjects x connections matrix. Subject-wise SC and FC were then correlated for all pairs of SC and FC (within and between subjects). On-diagonals of this matrix represent within-subject SC-FC (SC-SelfFC); off-diagonals represent between-subject SC-FC (SC-OtherFC).

We compared individual SC and FC within and between all subjects by using Pearson’s correlations in order to determine whether individual SC correlates best with its own individual FC. We constructed a matrix of size *N*_*SC*_ × *N*_*FC*_ (*N* = the number of subjects, *N*_*SC*_ = *N*_*FC*_). The diagonal of this matrix captures the intrasubject (within) SC-FC correlations; the off-diagonal represents the intersubject (between) SC-FC (see Figure 1 for a visualization of this SC-FC matrix). We corrected the *p* value of each correlation value in the resulting matrix for multiple comparisons by using FDR (Matlab function fdr_bky; Benjamini, Krieger, & Yekutieli, 2006). Note that associations between all individual SC and FC within and between all subjects was also performed via eigenvector correlations to complement the Pearson’s correlation method. This method is described in the Supporting Information (Zimmermann et al., 2018).

We conducted 1,000 bootstrapped means of SC-SelfFC correlations and 1,000 bootstrapped means of SC-OtherFC correlations (Matlab function bootstrp), plotting the two bootstrapped distributions against each other. To evaluate the statistical significance of the differences between the distributions, we subtracted the SC-OtherFC distribution from the SC-SelfFC distribution and constructed a 95% confidence interval on this difference distribution (Matlab function prctile). To confirm our results, we conducted several secondary analysis. First, we conducted a global signal regression in order to minimize the effects of global signal differences on individual SC-FC relationships. We also logarithmized SCs and transformed them to a Gaussian distribution by resampling (Honey et al., 2009) to correct for exponentially distributed connection weights. Last, we used only SC present connections, as indirect connections may have an unknown effect on FC (Honey et al., 2009).

### Subject Variability in SC and in FC

We examined variability across subjects in SC as well as in FC via PCA. The objective was to understand whether the lack of subject specificity of SC-FC in the Berlin, HCP Lausanne, and NKI Rockland datasets was due to a large portion of common variance in the connectomes across subjects overpowering existing individual differences. To this end, we decomposed the subject-wise SC matrix (SC connections × subjects) and FC matrix (FC connections × subjects) via the Matlab princomp function (subjects as variables). The breakdown of variability in SC as well as FC across subjects was thus ascertained. From the PCA, we obtained for each principal component (PC) the following: eigenvalues, principal component loadings per subject, and principal component scores per each connection. To determine the significance of the resulting eigenvalues, we generated null distributions of eigenvalues for each PC by permuting the SC and the FC 100 times (scrambled across connections and subjects) and performing PCA of the resulting matrices. A *p* value for each PC eigenvalue was obtained as the proportion of times that the permuted eigenvalue exceeded the obtained eigenvalue.

We also computed the age effect on connectome variability by calculating the correlation, via partial least squares, of age (age vector, size: subjects × 1) with the subjects’ principal coefficient loadings of the significant PCs (size: subjects × number of significant PCs). Partial least squares is a multivariate method comparable with canonical correlation in that it computes the relationship between two matrices via orthogonal latent variables (Krishnan, Williams, McIntosh, & Abdi, 2011; McIntosh & Lobaugh, 2004). The significance of the resulting correlations was assessed via permutation testing (*N* = 1,000) of the singular values from singular value decomposition of the two matrices. Reliability of each principal component subject loading to the latent variable was assessed via bootstrapping (*N* = 500). We thus were able to compute how age corresponded to the significant variance across subjects.

## RESULTS

### Subject Specificity of SC-FC Predictions

**Figure 2.**
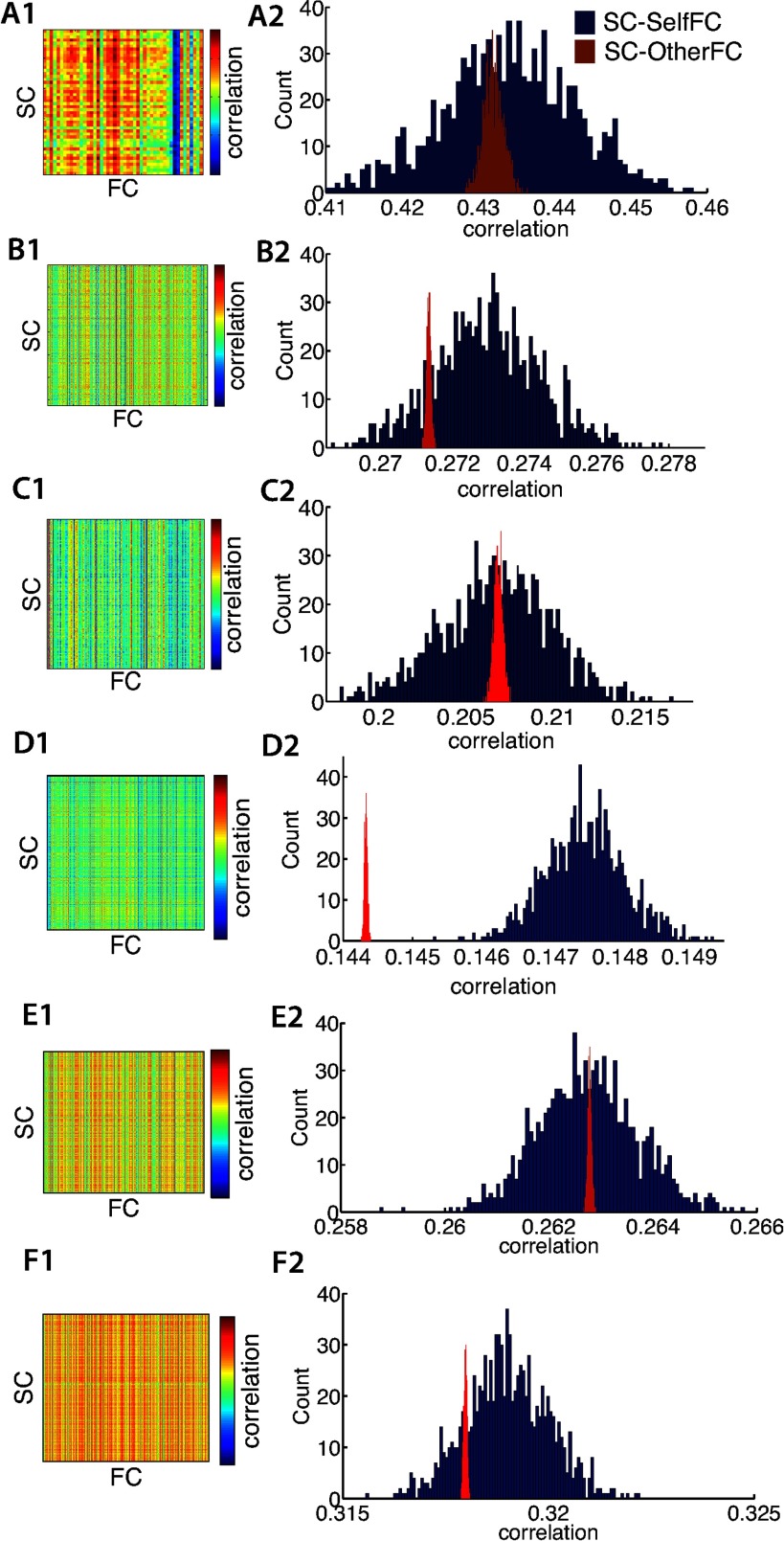
Bivariate Pearson’s correlations are shown for all combinations of SC and FC within and between subjects in the first column. Distribution histograms of bootstrapped means of intra- (SC-SelfFC) and inter- (SC-OtherFC) correlations are shown in the second column. Each row is a different dataset: A) Berlin B) HCP Lausanne C) Rockland D) HCP Glasser, E) HCP Destrieux F) HCP DK.

We first quantified the SC-FC relationship at the group-average level. The correlation between averaged SC and averaged FC was as follows: *r* = 0.59, 0.47, 0.41, 0.34, 0.40, 0.47, *p* < 0.001, for Berlin, HCP Lausanne, NKI, HCP Glasser, HCP Destrieux, and HCP DK, respectively. At the individual subject level, all subjects’ SCs were significantly correlated with all subjects’ FCs (between and within SC-FC; Pearson’s correlations, *p* < 0.001, FDR multiple comparison correction, *p* < 0.001). However, we found that SC-FC correlations were subject specific only for the HCP Glasser dataset, and not for the other datasets. This was assessed by comparing the bootstrapped within-subject SC-FC correlation distribution (SC-SelfFC) with the bootstrapped between-subject SC-FC correlation distribution (SC-OtherFC), as discussed in the Methods. See Figure 2 for subject specificity assessed using the simple bivariate correlation, and Supporting Information Figure S2 (Zimmermann et al., 2018) for subject specificity assessed using the eigenvector correlation approach. Means and CIs on the difference distributions are shown in Table 2 for simple correlations and Supporting Information Table S2 (Zimmermann et al., 2018) for eigenvector correlations. The results were consistent using the two approaches.

**Table 2.** Means and 95% CIs on the difference distribution, calculated as the difference between the SC-SelfFC and SC-OtherFC distributions

**Dataset**	**Simple correlation**	
	**Mean**	**CI**
Berlin	M = 0.0013	[−0.0169, 0.0190]
HCP, Lausanne	M = 0.0016	[−0.0012, 0.0044]
NKI Rockland	M = −5.8447e−04	[−0.0109, 0.0065]
HCP, Glasser	M = 0.0032	[0.002, 0.0043]*
HCP, Destrieux	M = −2.2348e−04	[−0.0019, 0.002]
HCP, DK	M = 0.001	[−0.0001, 0.0017]

* Significant subject specificity, whereby the distribution of intrasubject SC-FC was higher than the distribution of intersubject SC-FC.

In summary, we found that for all but the HCP Glasser dataset, a subject’s SC did not correlate better with its own FC than with another subject’s FC. These results remained consistent when using distance corrected SCs or only SC present connections. For the HCP Glasser dataset, the within-subject SC-FC was significantly higher than the between-subject SC-FC.

### Subject Variability in SC and in FC

**Figure 3.**
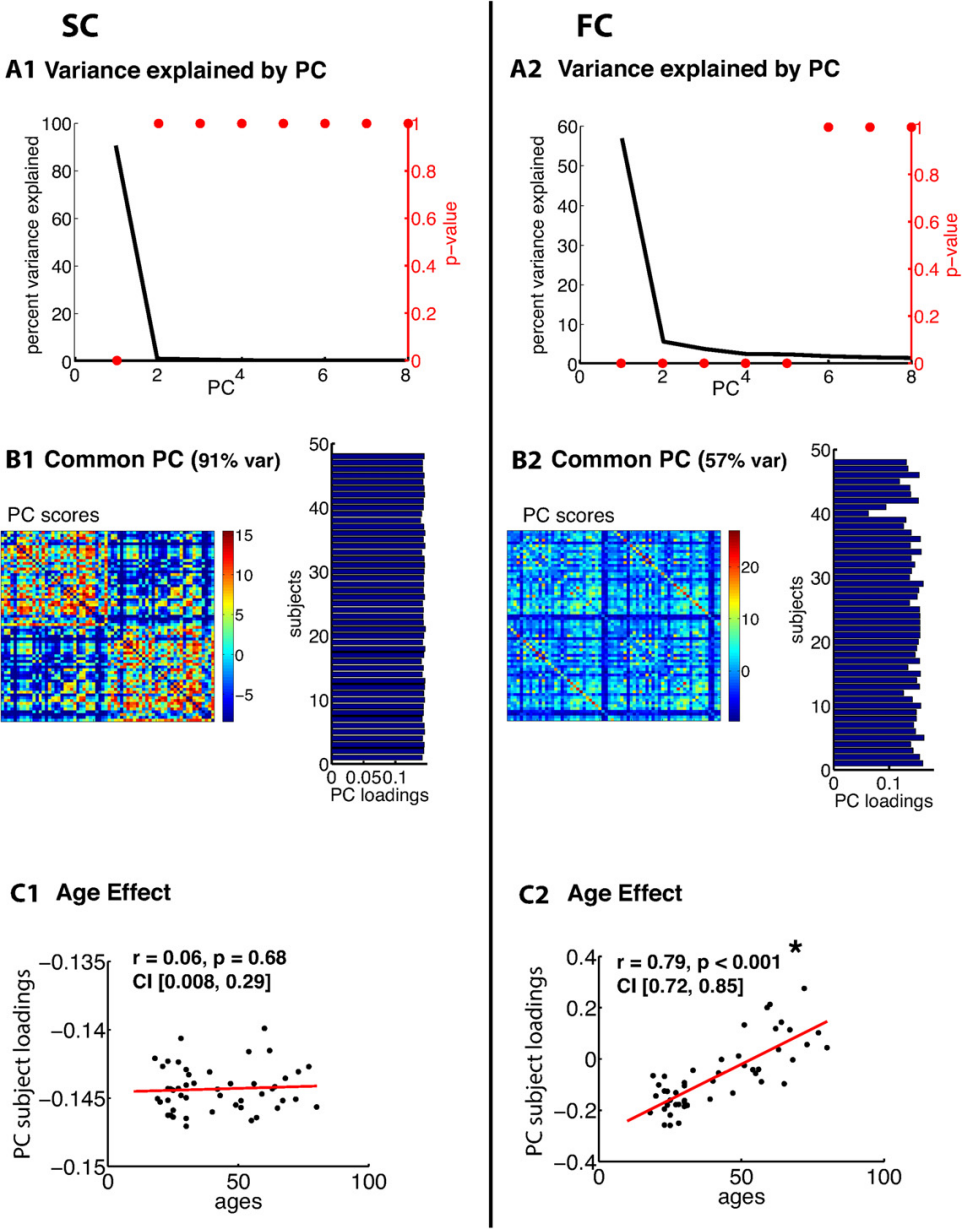
PCA of SC (left column) and FC (right column) for the Berlin dataset. (A1, 2) The first row depicts the percent of total variance explained for each PC with corresponding *p* values in red. (B1, 2) The second row shows the PC connectome scores as well as the individual subject loadings on the first PC (all subjects positively loaded). (C1, 2) The third row shows the effect of age.

**Figure 4.**
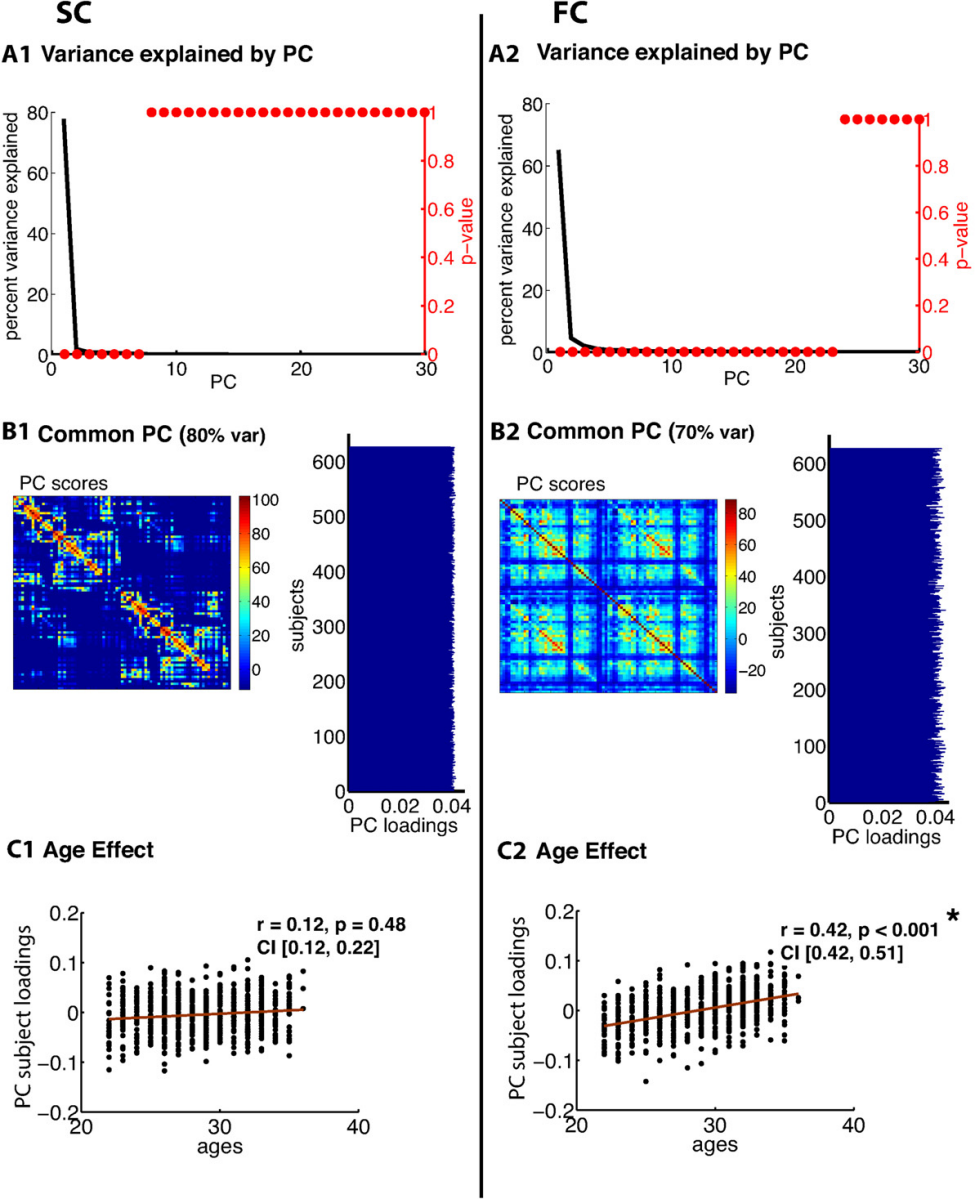
PCA of SC (left column) and FC (right column) for the HCP Lausanne dataset. (A1, 2) The first row depicts the percent of total variance explained for each PC with corresponding *p* values in red. (B1, 2) The second row shows the PC connectome scores as well as the individual subject loadings on the first PC (all subjects positively loaded). (C1, 2) The third row shows the effect of age.

**Figure 5.**
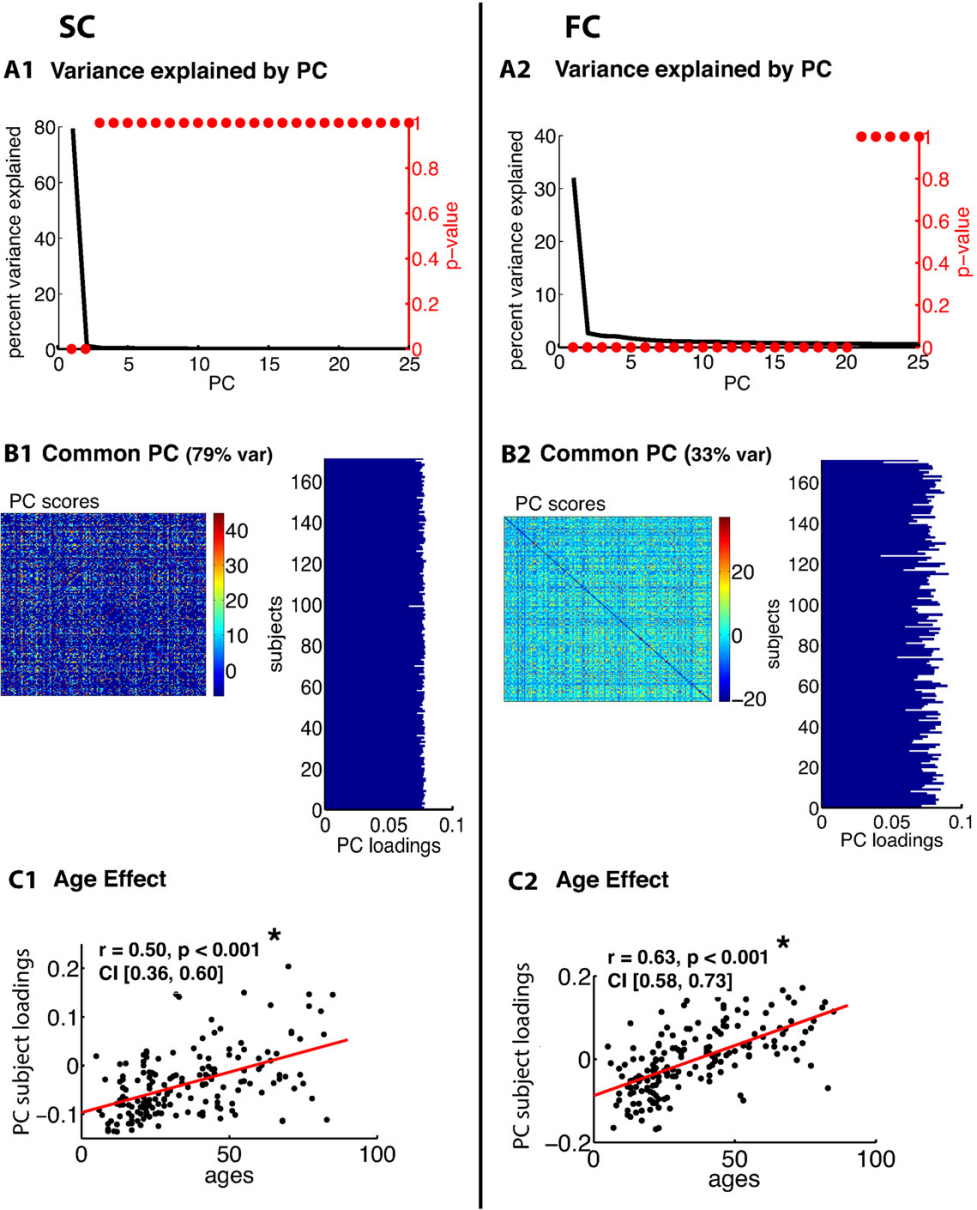
PCA of SC (left column) and FC (right column) for the Rockland dataset. (A1, 2) The first row depicts the percent of total variance explained for each PC with corresponding *p* values in red. (B1, 2) The second row shows the PC connectome scores as well as the individual subject loadings on the first PC (all subjects positively loaded). (C1, 2) The third row shows the effect of age.

**Figure 6.**
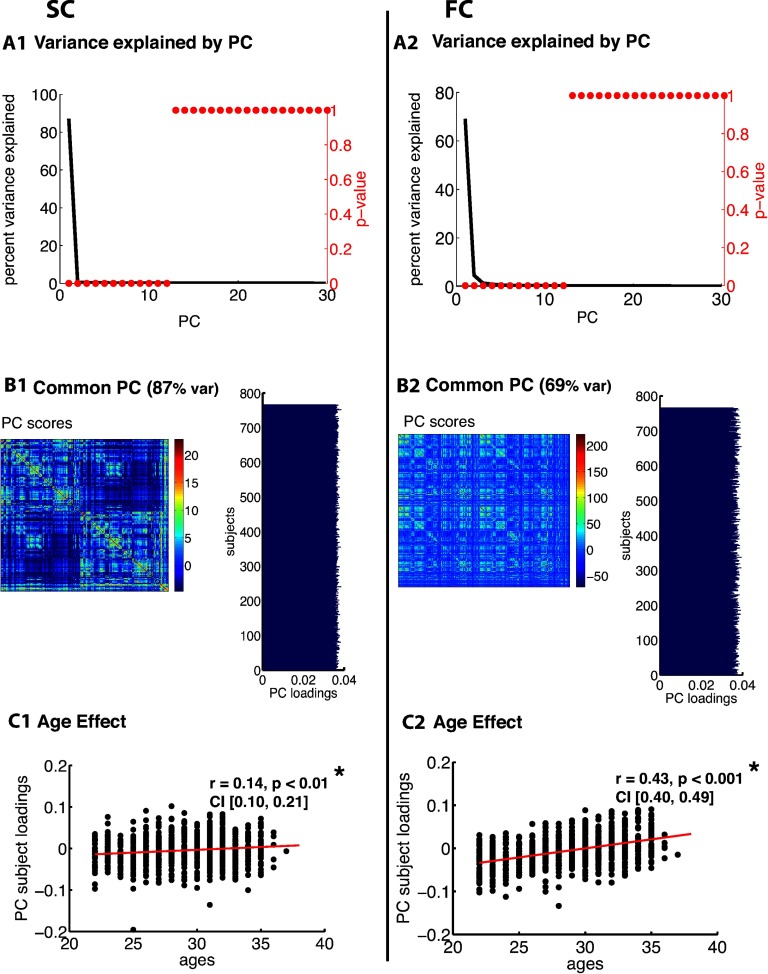
PCA of SC (left column) and FC (right column) for the HCP Glasser dataset. (A1, 2) The first row depicts the percent of total variance explained for each PC with corresponding *p* values in red. (B1, 2) The second row shows the PC connectome scores as well as the individual subject loadings on the first PC (all subjects positively loaded). (C1, 2) The third row shows the effect of age.

**Figure 7.**
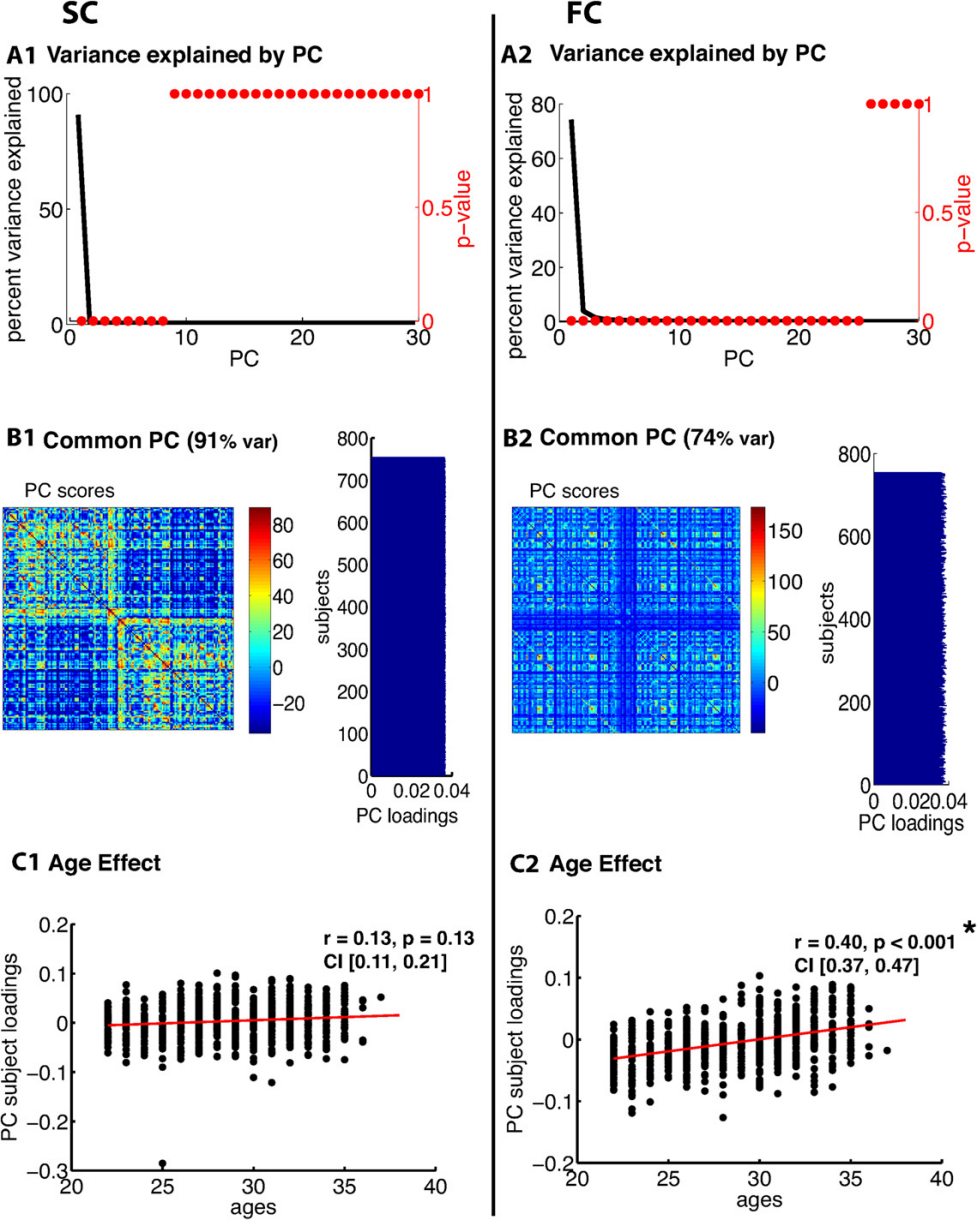
PCA of SC (left column) and FC (right column) for the HCP Destrieux dataset. (A1, 2) The first row depicts the percent of total variance explained for each PC with corresponding *p* values in red. (B1, 2) The second row shows the PC connectome scores as well as the individual subject loadings on the first PC (all subjects positively loaded). (C1, 2) The third row shows the effect of age.

**Figure 8.**
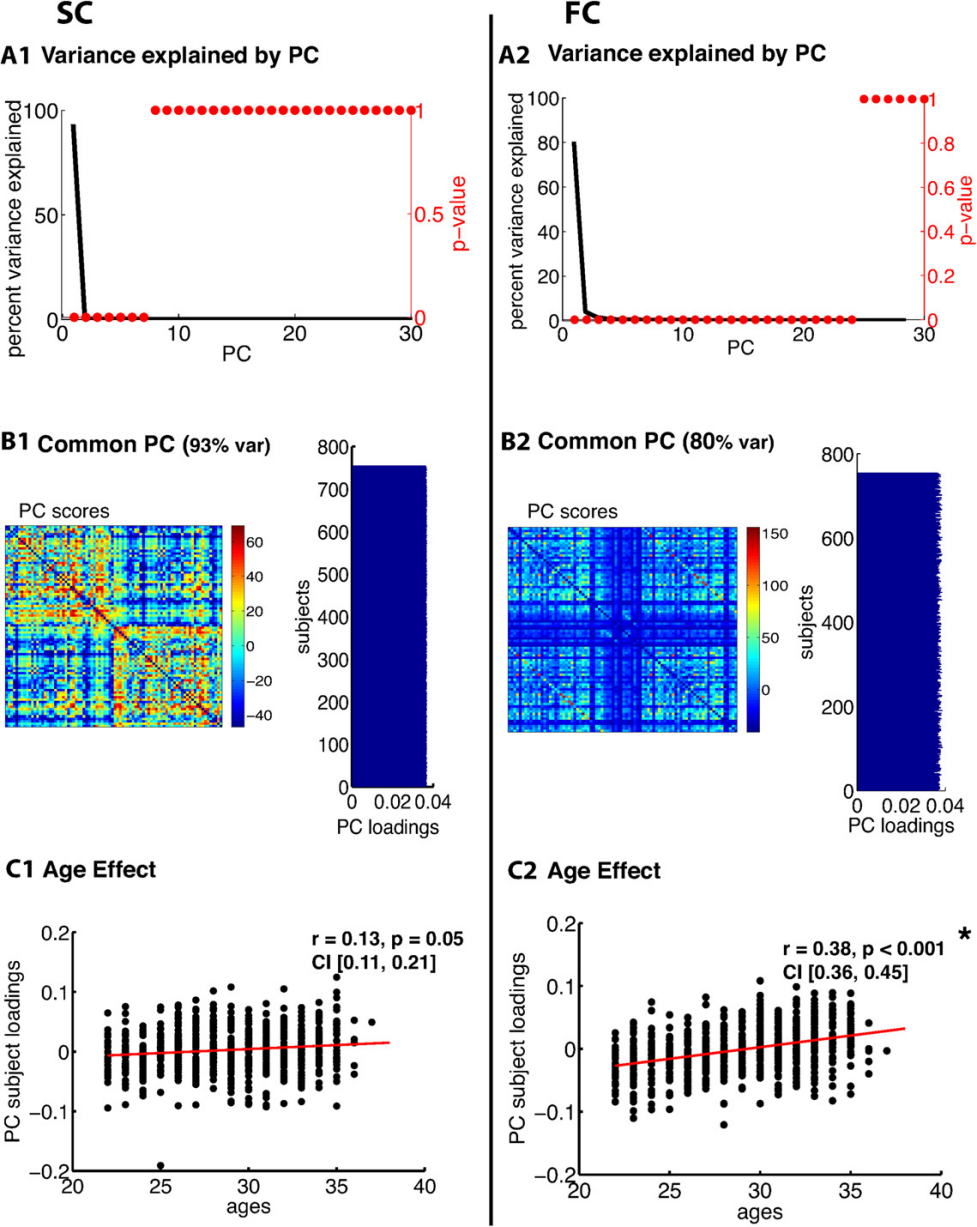
PCA of SC (left column) and FC (right column) for the HCP DK dataset. (A1, 2) The first row depicts the percent of total variance explained for each PC with corresponding *p* values in red. (B1, 2) The second row shows the PC connectome scores as well as the individual subject loadings on the first PC (all subjects positively loaded). (C1, 2) The third row shows the effect of age.

Figure 3, Figure 4, Figure 5, Figure 6, Figure 7, and Figure 8 show PCA results for Berlin, HCP Lausanne, NKI, HCP Glasser, HCP Destrieux, and HCP DK data, respectively. For both SC and FC across our datasets, the first component captured a very large portion of common variance across subject. All subjects loaded heavily on this common PC1; these principal component subject loadings are visualized in the bar plots on the right-hand side in Figures 3B–8B. The principal component scores (i.e., reconstructed matrix from PC1) for this common PC are visualized in the matrices on the left-hand side in Figures 3B–8B. These represent the features of the connectome that were captured by PC1. The variance explained by this first common PC was large in the SC (91, 80, 79, 91, and 93% variance explained for Berlin, HCP Lausanne, NKI, HCP Glasser, HCP Destrieux, and HCP DK datasets, respectively) and lower in the FC (57, 70, 33, 74, and 80% variance explained for Berlin, HCP Lausanne, NKI, HCP Glasser, HCP Destrieux, and HCP DK datasets, respectively). Eigenvalues for the first 30 PCs for all datasets are shown in Supporting Information Table S3 (Zimmermann et al., 2018). It is noteworthy that the HCP Glasser SC showed the largest number of significant principal components (HCP Glasser *N* = 12, Berlin = 1, HCP Lausanne = 7, NKI Rockland = 2, HCP Destrieux = 8, and HCP DK = 7).

A second pattern of results that we observed across all datasets was that SC was less variable than FC across subjects. There were fewer significant eigenvalues for the SC compared with the FC (see Supporting Information Table S3, Zimmermann et al., 2018). From the figures (Figures 3A–8A), the knee, or drop-off, in the variance explained by subsequent PCs (Cattell, 1966) was evidently sharper for the SC than the FC. Thus, although the common component was dominant for both modalities, the second and later components explained a larger portion of variance in the FC than in the SC.

Consistent with the above findings, we also noted differences between SC and FC in the strength of the age-related differences. We found an age effect in the FC for all six datasets (Berlin: *r* = 0.79, *p* < 0.001; HCP Lausanne: *r* = 0.42, *p* < 0.001; NKI: *r* = 0.63, *p* < 0.001; HCP Glasser: *r* = 0.43, *p* < 0.001; HCP Destrieux: *r* = 0.40, *p* < 0.001; and HCP DK: *r* = 0.38, *p* < 0.001). We found an age effect in the SC for two of the six datasets (Berlin [nonsignificant]: *r* = 0.06, *p* = 0.68; HCP Lausanne [nonsignificant]: *r* = 0.12, *p* = 0.48; NKI [significant]: *r* = 0.50, *p* < 0.001; HCP Glasser [significant]: *r* = 0.14, *p* = 0.035; HCP Destrieux [nonsignificant]: *r* = 0.13, *p* = 0.13; and HCP DK [nonsignificant]: *r* = 0.13, *p* = 0.05).

We compared brain volume across subjects to check for any age-related differences. For the Berlin and the Rockland dataset, tissue segmentation was performed and partial volume maps were derived using FSL FAST. Total brain volume was computed by summing the GM and WM tissue volumes. Total brain volume across subjects was correlated with region-wise SC (Berlin: *r* = 0.22, *p* = 0.14; Rockland: *r* = 0.18, *p* = 0.17) and FC (Berlin: *r* = 0.17, *p* = 0.31; Rockland: *r* = 0.09, *p* = 0.40); no effect was found. Volume differences in the HCP data were already accounted for via the FIX method.

Finally, it is noteworthy that our results remained robust following a number of secondary analyses. These are described in detail in the Methods, and include the following: global signal regression, including only SC present connections in the analyses, and logarithmizing and resampling SCs to a Gaussian distribution. Because results remained robust against these corrections, the results shown are those based on the original matrices. Please see Supporting Information Table S2 (Zimmermann et al., 2018) for the PCA results on logarithmized SCs redistributed to Gaussian.

## DISCUSSION

### Subject Specificity in SC-FC

Initial studies of SC-FC correspondence (Greicius et al., 2009; Honey et al., 2007; Honey et al., 2009; Koch et al., 2002) show that there is a relationship between these two entities via linear (Honey et al., 2009) as well as more complex methods (Misic et al., 2016). However, there remains a gap in our understanding of how the two measures are related at the individual level. In the present study, we showcase how individual SC corresponds with individual FC by using simple linear metrics in six separate datasets (Berlin, HCP Lausanne, NKI Rockland, HCP Glasser, HCP Destrieux, and HCP DK). The datasets differed in sample size, acquisition, and processing methods as well as age spectrums. The question was whether the correspondence of individual SC-FC matrices was greater than if two matrices were randomly paired.

Our results showed that although there is a correlation between group-averaged SC and FC, replicating previous findings (Greicius et al., 2009; Hermundstad et al., 2013; Honey et al., 2007; Honey et al., 2009; Koch et al., 2002; Misic et al., 2016; Ponce-Alvarez et al., 2015; Skudlarski et al., 2008; van den Heuvel et al., 2009), the specificity of this SC-FC relationship was not unique to an individual. Five of the datasets examined did not show subject specificity of the SC-FC correspondence, so that within-subject SC-FC did not exceed random pairings of SC-FC. This would suggest that individual SC cannot predict individual FC beyond chance. However, when the analysis was conducted on the HCP data with the Glasser parcellation, significant subject specificity was observed. This would suggest that while subject specificity assessed on standard datasets via standard parcellation and processing methods is difficult to ascertain, it may be obvious only when higher resolution data as well as finer parcellations are used.

Our finding that intrasubject SC-FC correspondence exceeded intersubject SC-FC correspondence for the HCP Glasser dataset, but not for the remaining datasets, supports the hypothesis by Honey et al. (2009). Honey et al. (2009) speculated that the individual SC-FC fit would be significant if shown on a large enough dataset of high fidelity. However, fidelity of the data will depend on a number of factors, including the quality and rigor of the data acquisition procedures, the processing methods (e.g., tractography), and the parcellation used. The acquisition procedures alone were unlikely to be the sole driving factor behind subject specificity, as these were consistent across the HCP data. We hypothesized that the superior subject specificity of the Glasser HCP data (compared with the HCP Lausanne) was due to the high-precision parcellation used (Glasser et al., 2016). However, these two HCP datasets also differed in the tractography method (probabilistic vs. deterministic). Thus we endeavored to reevaluate our findings post hoc by using two additional HCP datasets with probabilistic tractography processed in the same way as the Glasser HCP, except with different parcellation methods. We used the FreeSurfer convolution-based probabilistic Destrieux atlas (Destrieux et al., 2010) and the DK atlas (Desikan et al., 2006). We did not find subject specificity with the HCP Destrieux and the HCP DK, suggesting that the Glasser parcellation allows for a fitting of individual structure and function that could not otherwise be observed. The Glasser multimodal parcellation is based on functional properties with improved areal feature-based cross-subject alignment, rather than solely geometric and morphological properties. Thus, the method improves the neuroanatomical precision of individual parcellations. It is important to point out that despite the improvement, the HCP Glasser dataset was only slightly better than the others, and would not pass a direct head-to-head comparison since the presence of significance in one dataset and the absence of significance in another does not mean the two datasets are themselves significantly different.

### Subject Specificity in SC-FC is Limited by Variability Within Modality

The second set of findings showed that the unique portion of variance that exists in either modality alone is limited. This may restrict the portion of SC that can reasonably be captured by individual FC. We had hypothesized that the lack of subject specificity in the Berlin, HCP Lausanne, NKI Rockland, HCP Destrieux, and HCP DK dataset was due to a large portion of common variance in the connectomes across subjects that overpowered any existing individual differences. Indeed, our results confirmed that there is a large portion of common variance in SC across subjects. This was the case regardless of the sample size, data quality, or parcellation. Interestingly, even in the Glasser dataset, where SC-FC subject specificity was observed, the common component was strikingly large. We did observe, however, that SC variability was captured via a greater number of components in the Glasser dataset compared with the other datasets, suggesting greater interindividual differences in the SC. Although the smaller datasets (e.g., Berlin) generally had fewer SC components, the variability that was observed in the HCP Glasser SC was not merely due to sample size, as the HCP Destrieux and HCP DK datasets were comparable to the HCP Glasser dataset in terms of sample size.

We also observed a large common component in the FC across subjects. However, this component accounted for a smaller portion of total variance than the SC common component. Moreover, a smaller number of significant variance components were found in the FC across subjects compared with in the SC. Together, these results suggest that FC is more variable than SC across subjects. This can also be observed in the striping of the SC-FC correspondence matrix, where some FCs correlate strongly with all SCs, while others correlate very little with all SCs. Note that this does not mean that individual differences in SC were not observed, but rather that the variance in SC that maps onto the corresponding variance in FC is weaker than one may expect intuitively.

In the FC, a significant portion of variance was related to age, particularly for the two datasets with a wide age range (Berlin, NKI: age = 20–80, 5–85). This is consistent with previous reports of age effects on FC (Andrews-Hanna et al., 2007; Damoiseaux et al., 2007; Ferreira & Busatto, 2013; Sala-Llonch et al., 2014). Interestingly, age did not account for a significant portion of between-subjects variance in SC for four of the six datasets. We found an age effect in the SC only for the HCP Glasser dataset and the NKI Rockland dataset. In the NKI Rockland dataset, the large observed age effect in SC was likely a consequence of the wide age distribution and the inclusion of child subjects. The gray-white matter boundary is ill defined in children, and incomplete myelination results in weaker tractography-based estimation of SC (Deoni, Dean, Remer, Dirks, & O’Muircheartaigh, 2015; Thompson et al., 2005).

The limited amount of between-subjects variability in both SC and FC that we observed was comparable to findings by Marellec et al. (2016), where a large portion of variance was accounted for by an invariant core that was consistent across subjects (SC: ∼86%; FC: ∼59%). There it was shown that the invariant core of SC correlated with the invariant core of FC. Along the same lines, Waller et al. (2017) suggested that the specificity of connectome fingerprinting using FC was limited by the large amount of common variance across subjects.

The decomposition approach we used here was helpful for separating common and unique variance, and identifying aspects of the connectome that express each portion of variance. Data-driven classification algorithms like clustering are an alternate approach that can be used to express similarities and differences between-subject connectomes (Amico et al., 2017; Iraji et al., 2016). Recently, a consensus clustering algorithm has been introduced that can be helpful for identifying how aspects of the connectome are combined to express these intersubject similarities and differences (Rasero et al., 2017).

### Limitations on the Study of Variability Within Modality

The study of variability within SC and FC each faces its unique limitations. Variation in acquisition, processing, and connectome metrics as well as statistical methods may impact the extent of between-subjects variability observed. For instance, for SC, the diffusion method, tractography (Bonilha et al., 2015), SC metric (Buchanan, Pernet, Gorgolewski, Storkey, & Bastin, 2014), or ROI size (Bonilha et al., 2015) have been shown to affect variability and reproducibility of SCs. FC variability across subjects is affected by the choice of metric (Marrelec et al., 2016). For example, the amount of common variance may be slightly higher when using correlation compared with mutual information for the calculation of FC. On the other hand, the common component of FC that is invariant across subjects was comparable for dynamic and static FC (Marrelec et al., 2016). Nonetheless, the correlation between SC and FC may be limited by the dynamic fluctuation of FC on short time windows (Allen et al., 2014; Deco, Kringelbach, Jirsa, & Ritter, 2016; Hutchison et al., 2013). SCs may better correlate with temporally stable rsFC (Honey et al., 2009). To this end, we considered only SC present connections in a secondary analysis, as these have been shown to have more stable resting-state FC (Shen et al., 2015).

One important question is whether increased between-subjects variation in the FC is a consequence of nonneural influences such as vascular variability or head motion (Geerligs, Tsvetanov, Cam, & Henson, 2017) or reflects real, meaningful variability in neural activation. If meaningless between-subjects variability in FC can be reduced, FC has the best chance to be able to capture subtle individual differences in SC. In addition to the corrections described in the methods, FC between-subjects variability was minimized via a secondary global signal regression (GSR) analysis (Berlin dataset, NKI Rockland dataset). Yet, lack of SC-FC subject-specific correlation in five of the six persists despite these secondary analyses.

### Future Directions

Computational models that investigate how SC gives rise to FC may be particularly helpful for furthering our understanding of how individual SC and FC are linked (Jirsa, Sporns, Breakspear, Deco, & McIntosh, 2010; Kringelbach, McIntosh, Ritter, Jirsa, & Deco, 2015; Kunze, Hunold, Haueisen, Jirsa, & Spiegler, 2016; Ritter et al., 2013; Roy et al., 2014). The mechanisms by which individual FC comes about from individual SC may be the key to understanding subject-specific differences. To this end, parameters from generative models combining individual empirical SC and FC can be used (Schirner, McIntosh, Jirsa, Deco, & Ritter, 2018). Variability in these parameters have already been shown to be useful for revealing individual differences relevant for cognition (Falcon et al., 2016; Falcon et al., 2015; J. Zimmermann et al., 2018). These parameters may even exceed the predictive capacity of individual connectomes (Zimmermann et al., 2018).

## ACKNOWLEDGMENTS

Data were provided (in part) by the Human Connectome Project, WU-Minn Consortium (Principal Investigators: David Van Essen and Kamil Ugurbil; 1U54MH091657), funded by the 16 NIH Institutes and Centers that support the NIH Blueprint for Neuroscience Research, and by the McDonnell Center for Systems Neuroscience at Washington University. The authors gratefully acknowledge the computing time granted by the John von Neumann Institute for Computing (NIC) and provided on the supercomputer JURECA at Jülich Supercomputing Centre (JSC) (www.fz-juelich.de, Grant NIC 8344 and NIC 10276 to P.R.).

## AUTHOR CONTRIBUTIONS

Joelle Zimmermann: Conceptualization; Data curation; Formal analysis; Funding acquisition; Investigation; Methodology; Project administration; Software; Visualization; Writing – original draft. Petra Ritter: Data curation; Funding acquisition; Writing – review & editing. Michael Schirner: Data curation; Writing – review & editing. John Griffiths: Data curation. Randy McIntosh: Funding acquisition; Supervision; Writing – review & editing.

## FUNDING INFORMATION

Petra Ritter, John von Neumann Institute for Computing, Award ID: 8344 & 10276. Randy McIntosh, NSERC, Award ID: RGPIN-2017-06793. Petra Ritter, German Ministry of Education and Research: US-German Collaboration in Computational Neuroscience, Award ID: 01GQ1504A. Petra Ritter, German Ministry of Education and Research: Bernstein Focus State Dependencies of Learning, Award ID: 01GQ0971-5. Petra Ritter, European Union Horizon 2020, Award ID: ERC Consolidator Grant BrainModes 683049. Petra Ritter, Stiftung Charité/Private Exzellenzinitiative Johanna QuandtPetra Ritter, Berlin Instititute of Health (BIH Johanna Quandt Professorship for Brain Simulation).

## TECHNICAL TERMS

Structural connectivity: The physical pathways connecting regions in the brain; inferred from diffusion MRI and tractography.
Functional connectivity: The temporal co-activations between regions in the brain; derived by calculating all pairwise correlations of regional time series of resting-state fMRI activity.
Subject specificity of SC-FC: The finding that an SC correlates better with its corresponding FC (within-subject) than random pairing with another subject’s FC (between-subject).
Eigenvector correlations: Calculated as the maximum Pearson’s correlation between all significant eigenvectors from PCA decomposed individual SC and decomposed individual FC.
SC-SelfFC: Within-subject SC-FC Pearson’s correlations; SC and FC are from different subjects.
SC-OtherFC: Between-subject SC-FC Pearson’s correlations; SC and FC are from the same subject.
Principal component scores: Scores from PCA; here these represent the weights of individual connections toward the pattern identified by each principal component.
Principal component subject loadings: Loadings from PCA; here these represent the loadings of individual subjects towards the pattern identified by each principal component.
