# Supplementary material for: Subject specificity of the correlation between large-scale structural and functional connectivity
Source: Netw Neurosci. 2018 Oct 1;3(1):90–106. doi: 10.1162/netn_a_00055 (PMC6326745; doi:10.1162/netn_a_00055)
Supplement: Supplementary file 1 [file netn-03-90-s001.pdf]

Zimmermann, J., Griffiths, J., Schirner, M., Ritter, P., & McIntosh, A.R. (2018). Supporting Information for "Subject-specificity of the correlation between large-scale structural and functional connectivity." *Network Neuroscience*, *3*(1), 90-106. https://doi.org/10.1162/netn_a_00055

## Supplementary Materials

Table S1. Structural, diffusion and rsfMRI imaging acquisition parameters for 3 datasets

| Scanner | Structural Acquisition | Diffusion Acquisition | rsfMRI Acquisition |
|---|---|---|---|
| **Human Connectome Project**<br>*Van Essen et al. (2013), Ugurbil et al. (2013), Sotiropoulos et al. (2013)* | | | |
| 3T Siemens Skyra, 32-channel head coil | *T1w (3D MPRAGE):* Acquisition time = 7:40, TR = 2400, TE = 2.14, FA = 8 $^{o}$, FoV = 224x224mm, voxel size = 0.7mm, BW = 210Hz/Px<br>*T2w (3D T2-SPACE):* Acquisition time = 8:24, TR = 3200, TE = 565, FA = variable, FoV = 224x224, 0.7mm isotropic voxels, BW = 744Hz/Px | Spin-echo EPI, TR = 5520ms, TE = 89.5, FA = 78 $^{o}$, refocused FA = 160 $^{o}$, FoV = 210x180, matrix = 168x144, slice thickness 1.25mm, 111 slices, 1.25mm isotropic voxels, multiband factor = 3, echo spacing = 0.78ms, BW = 1488 Hz/Px, b-values 1000, 2000, 3000 s/mm$^2$ | Gradient-echo EPI, Acquisition time = 14:33 mins, TR (repetition time) = 720 ms, TE (echo time) = 33.1 ms, flip angle (FA) = 52$^{o}$, field of view (FoV) = 208 x 180mm, 72 slices, 2mm isotropic voxels, Multiband = 8, echo spacing = 0.58ms, BW = 2290Hz/Px |
| **Berlin**<br>*Ritter et al. (2013)* | | | |
| 3T Siemens Tim Trio, 12-channel head coil | *T1w (MPRAGE):* TR = 1900 ms, TE = 2.25ms, FA = 98 $^{o}$, FoV 230, 192 sagittal slices, 0.9x0.9x0.9mm voxel slize, 1mm slice thickness<br>*T2w:* Acquisition time = 5:52, TR = 5000ms, TE = 502ms, voxel size 1x1x1mm, FoV = 256mm | 61 transversal (2 mm thick) slices, TR = 7500ms, TE = 86ms, FoV = 220mm, 96 matrix, 2.3 x 2.3 x 2.3 mm voxels, 64 diffusion gradient directions with b-values of 1000 s/mm2, | EPI, Acquisition time = 22 mins, 661 volumes acquired consisted of 32 transversal (3 mm thick) slices, TR = 1940 ms, TE = 30 ms, FA = 78$^{o}$, FoV = 192 mm, 64 matrix, 3x3x3 mm isotropic voxels |
| **NKI Rockland**<br>*Brown et al. (2012)*<br>*http://fcon_1000.projects.nitrc.org/indi/pro/nki.html* | | | |

| | | | |
|---|---|---|---|
| Siemens Trio 3T | *T1w (MPRAGE):* Acquisition time 10:42, TR = 2500ms, TE = 3.5ms, 192 slices with voxel size $1x1x1mm^3$ *T2w:* Acquisition time = 0:15, TR = 2500ms, TE = 11ms, FoV = 216mm, slice thickness = 3mm, 38 transversal slices, voxel size = 3x3x3mm | Acquisition time = 13:32, TR = 10000 ms, TE = 91ms, 58 slices with voxel size 2x2x2mm along 64 diffusion weighted gradients, b = 1000 $s/mm^2$ | EPI, Acquisition time = 10:55, TR = 2500 ms, TE = 30 ms, 38 slices with 3x3x3mm voxel size |

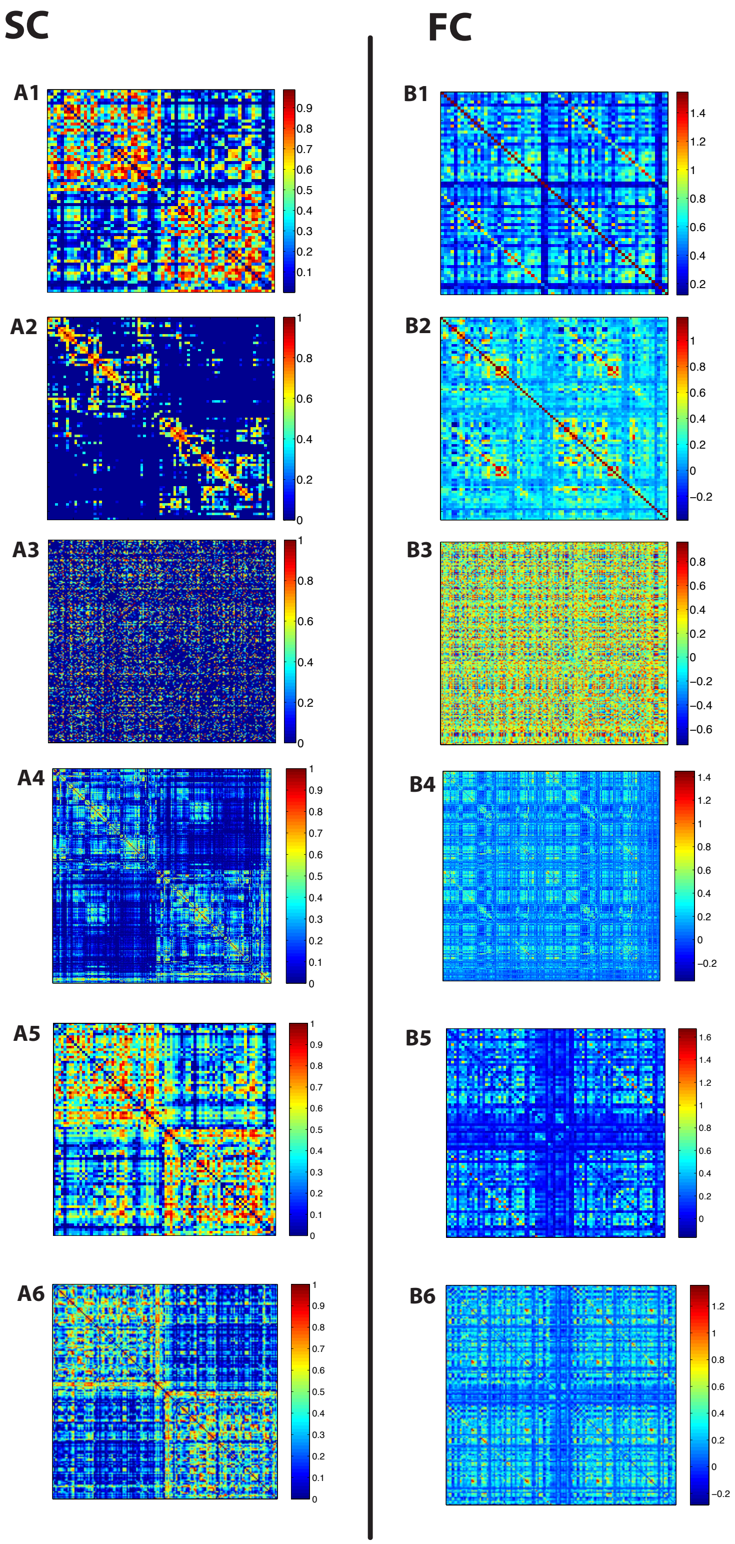


Figure S1. Representative SC (A, left column) and FC (B, right column) for 1) Berlin dataset 2) HCP Lausanne dataset 3) NKI dataset, 4) HCP Glasser dataset 5) HCP DK dataset 6) HCP Destrieux dataset

**Eigenvector correlations**

To examine the SC-FC relationship, in addition to Pearson's correlations between SC and FC, we use a second ("eigenvector correlation") method where we decompose individual connectomes and compare the components of the structural and functional connectomes that share the greatest amount of common variance between the modalities. This is particularly important because the patterns of similarity between individual SC and FC may not be manifested across the whole connectome (as is assumed by the Pearson's correlation method), but rather in particular components of the connectome.

The approach we used is similar to that visualized by Deco and colleagues for model prediction of empirical data in their Figure 4 (G. Deco et al., 2014). That is, each individual's SC was first decomposed via PCA (Matlab pcacov). The same was done for each individual's FC. Thus, for each individual SC and each individual FC we obtained PC eigenvalues and corresponding eigenvectors (Matlab output COEFF from function pcacov). We used permutation testing to select significant PCs for further analysis. This was done by permuting the SC matrix and the FC matrix 100 times (scrambled across connections) and performed PCA of the resulting matrices to generate null distributions of eigenvalues for each PC. A p-value for each PC eigenvalue was obtained as the proportion of times that the permuted eigenvalue exceeded the obtained eigenvalue for a component in that ordinal position. Significant PCs ($p < 0.05$) were retained for further analysis. The retained eigenvectors from the individual SC (vector $N_{PC\_SC}$) were correlated with all retained eigenvectors from the individual FC (vector $N_{PC\_FC}$), resulting in a $N_{PC\_SC} * N_{PC\_FC}$ matrix. The maximum correlation was then selected (i.e., the maximum of the $N_{PC\_SC} * N_{PC\_FC}$ eigenvector correlation matrix). The eigenvectors that produced this maximum correlation therefore represented the aspects of the SC and FC connectomes from the decomposition that were maximally correlated. This analysis was done for all within-subject SC-FC and between subject SC-FC; that is, all subject SCs were compared against all subject FCs. The resulting correlations were plotted, constructing a $N_{SC}$ x $N_{FC}$, with the diagonal representing SC-FC correlations where SC and FC were from the same subject.

The results from Table S2 and Figure S2, where associations between SC-SelfFC and SC-OtherFC were calculated as eigenvector correlations were consistent with the findings using Pearson's correlations. The distribution of SC-SelfFC correlations exceeded SC-OtherFC correlations only within the Glasser dataset.

Table S2. Mean and 95% CIs of the difference distribution calculated as the difference between the SC-SelfFC distribution and SC-OtherFC distribution, where SC-FC are calculated as eigenvector correlations. The * indicates a significant subject-specificity so that the distribution of intra-subject SC-FC is higher than the distribution of inter-subject SC-FC.

| Dataset | Eigenvector correlation | |
|---|---|---|
| | Mean | CI |
| **Berlin** | M = 0.0098 | [-0.0152, 0.0345] |
| **HCP, Lausanne** | M = 0.003 | [-0.0023, 0.0081] |
| **NKI Rockland** | M = -0.0015 | [-0.0217, 0.0165] |
| **HCP, Glasser** | M = 0.015 | [0.012, 0.019] * |
| **HCP, Destrieux** | M = 0.002 | [-0.0047, 0.0091] |
| **HCP, DK** | M = 0.0012 | [-0.0044, 0.0071] |

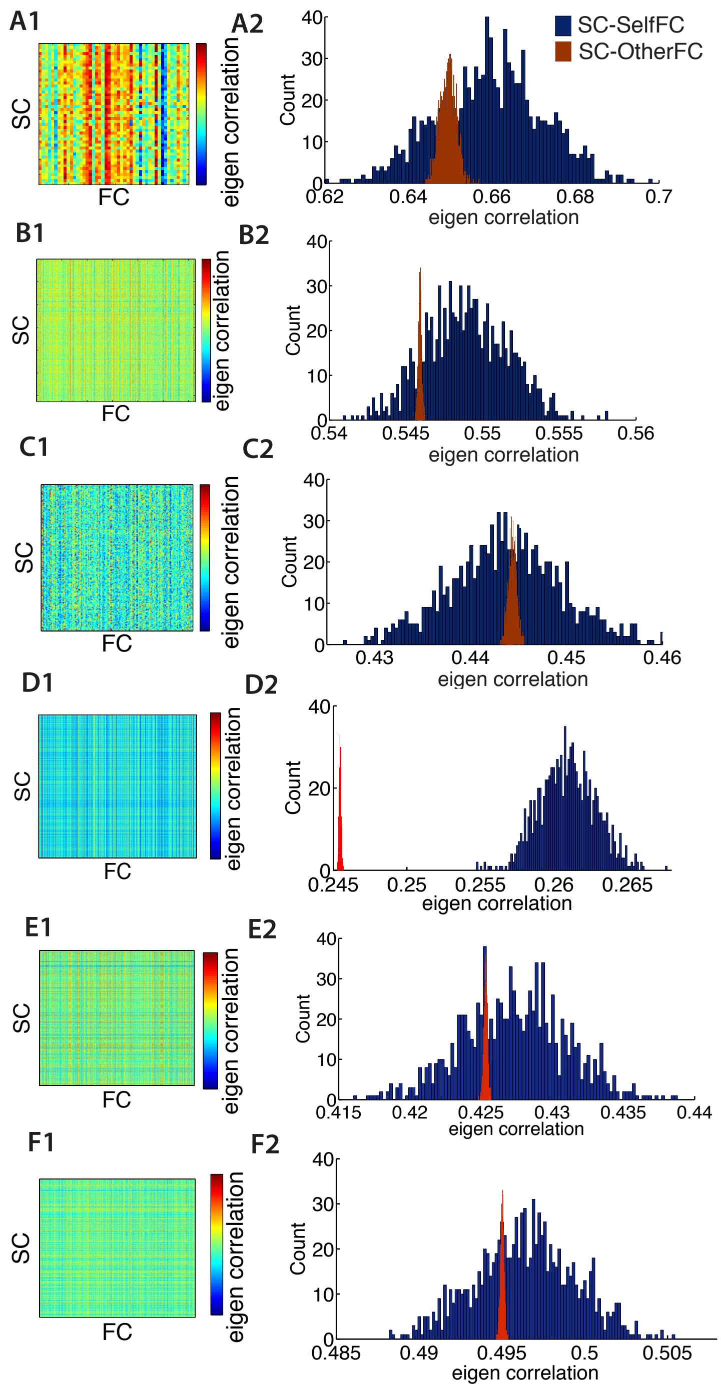


Figure S2. Eigenvector SC-FC correlations are shown for all combinations of SC and FC within and between subjects in the first column. These are the maximum correlations between individual SC eigenvectors and individual FC eigenvectors for all combinations of SC and FC within and between subjects (See Methods). Distribution histograms of bootstrapped means of intra (SC-SelfFC) and inter (SC-OtherFC) correlations are shown in the second column. Each row is a different dataset: **A)** Berlin **B)** HCP Lausanne **C)** Rockland **D)** HCP Glasser, **E)** HCP Destrieux **F)** HCP DK.

Table S3. Eigenvalues of the first 30 principal components from PCA of all subjects' SC and FC. Results of the analyses from the logarithmized and resampled to Gaussian SCs are shown for completeness (SC log & gaus). Significant (determined by permutation, see Methods) eigenvalues are shown in bold. Note that the first PC in all datasets represents the component that is common across subjects. The remaining PCs show variance across subjects.

| | **Berlin** | | | **HCP Lausanne** | | | **NKI** | | |
|---|---|---|---|---|---|---|---|---|---|
| **PC** | **SC** | **SC log gaus** | **FC** | **SC** | **SC log gaus** | **FC** | **SC** | **SC log gaus** | **FC** |
| **Common** | **43.53** | **42.47** | **27.33** | **503.68** | **501.22** | **437.86** | **135.74** | **134.60** | **56.29** |
| | 0.56 | 0.53 | **2.73** | **10.91** | **11.90** | **29.35** | **2.29** | **1.97** | **4.50** |
| | 0.40 | 0.39 | **1.84** | **4.98** | **4.78** | **13.15** | 1.08 | 0.91 | **3.63** |
| | 0.23 | 0.29 | **1.23** | **4.49** | **4.50** | **7.47** | 1.00 | 0.86 | **3.46** |
| | 0.21 | 0.25 | **1.18** | **3.53** | **3.33** | **4.93** | 0.92 | 0.77 | **2.84** |
| | 0.20 | 0.23 | 0.95 | **3.10** | **3.11** | **4.55** | 0.71 | 0.63 | **2.42** |
| | 0.19 | 0.21 | 0.82 | **1.75** | **1.90** | **3.83** | 0.69 | 0.61 | **2.08** |
| | 0.15 | 0.20 | 0.75 | 1.48 | 1.44 | **3.26** | 0.66 | 0.58 | **1.90** |
| | 0.14 | 0.18 | 0.73 | 1.39 | 1.40 | **3.03** | 0.61 | 0.52 | **1.84** |
| | 0.13 | 0.16 | 0.64 | 1.27 | 1.28 | **2.97** | 0.54 | 0.50 | **1.75** |
| | 0.12 | 0.15 | 0.58 | 1.16 | 1.20 | **2.90** | 0.51 | 0.48 | **1.73** |
| | 0.11 | 0.14 | 0.53 | 0.99 | 1.00 | **2.59** | 0.50 | 0.45 | **1.57** |
| | 0.11 | 0.13 | 0.51 | 0.95 | 0.99 | **2.56** | 0.48 | 0.44 | **1.47** |
| | 0.10 | 0.13 | 0.46 | 0.91 | 0.95 | **2.39** | 0.47 | 0.43 | **1.43** |
| | 0.09 | 0.12 | 0.43 | 0.87 | 0.90 | **2.26** | 0.44 | 0.42 | **1.41** |
| **Variance** | 0.09 | 0.11 | 0.42 | 0.85 | 0.88 | **2.17** | 0.39 | 0.37 | **1.28** |
| | 0.09 | 0.11 | 0.40 | 0.84 | 0.85 | **2.05** | 0.38 | 0.37 | **1.19** |
| | 0.08 | 0.11 | 0.38 | 0.78 | 0.80 | **1.89** | 0.37 | 0.36 | **1.19** |
| | 0.08 | 0.10 | 0.35 | 0.77 | 0.78 | **1.85** | 0.37 | 0.34 | **1.16** |
| | 0.07 | 0.10 | 0.34 | 0.73 | 0.76 | **1.77** | 0.35 | 0.34 | **1.14** |
| | 0.07 | 0.10 | 0.34 | 0.72 | 0.74 | **1.69** | 0.34 | 0.32 | 1.08 |
| | 0.07 | 0.09 | 0.32 | 0.71 | 0.73 | **1.68** | 0.33 | 0.32 | 1.05 |
| | 0.07 | 0.09 | 0.30 | 0.70 | 0.71 | **1.58** | 0.32 | 0.32 | 1.03 |
| | 0.07 | 0.09 | 0.30 | 0.66 | 0.70 | 1.52 | 0.31 | 0.31 | 1.03 |
| | 0.06 | 0.08 | 0.28 | 0.66 | 0.69 | 1.47 | 0.30 | 0.30 | 1.01 |
| | 0.06 | 0.08 | 0.28 | 0.65 | 0.67 | 1.45 | 0.30 | 0.29 | 0.98 |
| | 0.06 | 0.08 | 0.26 | 0.63 | 0.65 | 1.37 | 0.29 | 0.29 | 0.95 |
| | 0.06 | 0.08 | 0.24 | 0.60 | 0.63 | 1.34 | 0.28 | 0.29 | 0.93 |
| | 0.05 | 0.08 | 0.23 | 0.59 | 0.58 | 1.30 | 0.27 | 0.28 | 0.90 |
| | 0.05 | 0.07 | 0.21 | 0.58 | 0.57 | 1.26 | 0.27 | 0.27 | 0.89 |

| | **HCP Glasser** | | | **HCP Destrieux** | | | **HCP DK** | | |
|---|---|---|---|---|---|---|---|---|---|
| **PC** | **SC** | **SC log gaus** | **FC** | **SC** | **SC log gaus** | **FC** | **SC** | **SC log gaus** | **FC** |

| **Common** | **666.90** | **617.94** | **529.08** | **685.18** | **661.38** | **559.80** | **703.08** | **696.19** | **605.20** |
|---|---|---|---|---|---|---|---|---|---|
| | **3.37** | **3.78** | **35.21** | **2.97** | **3.23** | **30.14** | **2.34** | **2.23** | **30.04** |
| | **3.04** | **3.51** | **10.47** | **2.81** | **3.14** | **12.12** | **2.04** | **2.08** | **12.07** |
| | **2.06** | **2.32** | **6.83** | **1.87** | **2.45** | **5.97** | **1.64** | **1.78** | **5.66** |
| | **1.94** | **2.20** | **4.67** | **1.57** | **1.86** | **4.65** | **1.54** | **1.60** | **4.19** |
| | **1.62** | **2.06** | **3.61** | **1.45** | **1.74** | **3.73** | **1.37** | **1.39** | **4.12** |
| | **1.54** | **1.87** | **2.98** | **1.32** | **1.57** | **3.27** | **1.16** | **1.19** | **3.21** |
| | **1.48** | **1.69** | **2.84** | **1.26** | **1.48** | **2.97** | 1.13 | 1.17 | **2.87** |
| | **1.46** | **1.63** | **2.45** | 1.23 | 1.45 | **2.62** | 0.99 | 1.00 | **2.50** |
| | **1.37** | **1.58** | **2.26** | 1.12 | 1.31 | **2.49** | 0.90 | 0.93 | **2.30** |
| | **1.24** | **1.48** | **2.08** | 1.09 | 1.22 | **2.15** | 0.87 | 0.90 | **2.06** |
| | **1.15** | **1.32** | **2.00** | 0.98 | 1.09 | **1.94** | 0.79 | 0.79 | **1.94** |
| | 1.03 | **1.28** | 1.90 | 0.94 | 1.05 | **1.84** | 0.77 | 0.77 | **1.81** |
| | 0.98 | 1.17 | 1.82 | 0.92 | 1.02 | **1.73** | 0.70 | 0.72 | **1.71** |
| | 0.96 | 1.11 | 1.76 | 0.88 | 0.99 | **1.70** | 0.69 | 0.70 | **1.58** |
| **Variance** | 0.89 | 1.08 | 1.70 | 0.76 | 0.94 | **1.64** | 0.64 | 0.67 | **1.53** |
| | 0.84 | 0.96 | 1.66 | 0.73 | 0.87 | **1.58** | 0.60 | 0.66 | **1.46** |
| | 0.80 | 0.92 | 1.60 | 0.66 | 0.77 | **1.55** | 0.57 | 0.60 | **1.37** |
| | 0.76 | 0.90 | 1.54 | 0.62 | 0.75 | **1.45** | 0.56 | 0.57 | **1.33** |
| | 0.75 | 0.88 | 1.49 | 0.60 | 0.71 | **1.38** | 0.54 | 0.55 | **1.26** |
| | 0.69 | 0.83 | 1.43 | 0.57 | 0.67 | **1.34** | 0.52 | 0.54 | **1.22** |
| | 0.67 | 0.81 | 1.40 | 0.55 | 0.65 | **1.29** | 0.48 | 0.50 | **1.19** |
| | 0.64 | 0.78 | 1.31 | 0.54 | 0.63 | **1.24** | 0.47 | 0.49 | **1.17** |
| | 0.62 | 0.74 | 1.31 | 0.54 | 0.59 | **1.20** | 0.43 | 0.47 | **1.10** |
| | 0.60 | 0.74 | 1.24 | 0.48 | 0.58 | **1.16** | 0.42 | 0.44 | **1.06** |
| | 0.59 | 0.70 | 1.22 | 0.47 | 0.53 | **1.14** | 0.41 | 0.44 | 0.99 |
| | 0.57 | 0.69 | 1.19 | 0.45 | 0.52 | 1.10 | 0.40 | 0.42 | 0.98 |
| | 0.56 | 0.67 | 1.16 | 0.43 | 0.51 | 1.08 | 0.40 | 0.40 | 0.97 |
| | 0.53 | 0.66 | 1.14 | 0.41 | 0.50 | 1.04 | 0.38 | 0.39 | 0.90 |
| | 0.53 | 0.65 | 1.08 | 0.40 | 0.47 | 1.02 | 0.37 | 0.38 | 0.88 |
